# Medical and Philosophical Intelligence

**Published:** 1819-04

**Authors:** 


### MEDICAL AND PHILOSOPHICAL INTELLIGENCE.

*ROYAL SOCIETY.*—January 14, a paper on the *Corpora Lutea* was read by Sir EVERARD HOME. We shall at present state the principal facts related by the author, deferring the particular consideration of his Memoir until it shall be published in the Transactions.

The ovarium is loose and open in its texture previous to the age of puberty, when it becomes more firm and compact, and the corpora lutea make their appearance. In the cow, those bodies form a mass of convolutions, which Sir Everard compares to those of the brain. The ova are then formed in the lutea before and independently of sexual intercourse, but impregnation is necessary to their expulsion; and, on that taking place, they burst by extravasated blood; their cavities, after the escape of the ova, being always found distended with coagulated blood.

---

*Royal College of Surgeons.*—Feb. 15, the annual *Hunterian Oration* was delivered by Mr. ABERNETHY. It would be as impossible to give an adequate view of its scientific relation, as it would be to convey the most remote idea of its oratorial excellence, from mere auricular intelligence; and we are pleased to find that we may be spared the attempt, as it will shortly issue from the press: when in that form, we shall introduce it to our readers.

*An Improvement in the Form of the Catheter.*—An alteration from the ordinary form of the catheter, the idea of which would appear to be derived from Mr. Charles Bell's sound, that, from opinion, would appear to be of practical utility, has been proposed by Dr. M'Sweeny.

"This catheter," says Dr. M'S. "is twelve inches long; it has a bulbous extremity, and the rest of it is small in proportion to the bulb. There is a pyriform depression at the extremity of the bulb, in which depression the perforation is. The stilet is about an inch or more longer than the catheter, and has a pyriform knob at one extremity to correspond exactly with the depression at the end of the bulb. The other end of the stilet is not furnished with a ring. The stilet is put into the catheter at the bulbous extremity, and the pyriform knob fits the depression so exactly, that the catheter appears to have no orifice for the urine to escape. The stilet being about an inch longer than the catheter, it can be pushed up when the catheter is introduced. The act of pushing up the stilet will make the pyriform knob at its extremity rise out of the depression in the catheter, and the urine will flow freely out. The full flow of it may be suddenly stopped by pulling down the stilet to its former situation. In cases of calculi, the act of pushing up the stilet may be of service in keeping away concretions from the neck of the bladder when the urine is flowing."

The unpleasant effects sometimes,—that is, in cases of great irritability of the urethra,—arising from the roughness of the sides of those catheters which are perforated near the extremity with several small holes, and the insinuation of the membrane of the urethra into the calibre of the instrument, in those which have one large opening near the point, Dr. M'Sweeny also observes, will be thus avoided. The peculiar good properties of Mr. Bell's sound will, it is obvious, be also possessed by this instrument.*

---

The *high operation* for the stone in the bladder has recently been introduced into practice at Paris, by M. Souberbielle, with extraordinary success; such, indeed, as should draw the serious attention of surgeons again to this mode of operating. It was performed twenty-seven times by M. S. in the space of one year, on male subjects, of from fifty to eighty-six years of age, except in one instance, where the patient was only fifteen. The operations were executed according to the method of Friar Cosmo; making the incision in the bladder from within outwardly, without having previously distended it by injections or retention of the urine. Mr. Carpue witnessed this operation twice at Paris, and is said to think very favourably of its propriety in many cases.

The same method of performing lithotomy was a few months since performed at St. George's Hospital, by Sir Everard Home,

* Catheters of this kind may be obtained from Still, surgical instrument maker, Leicester-street.

with the most favourable results, on a boy seven years of age: but, as some of the minor steps of the operation were effected in a peculiar manner, we shall detail the principal circumstances respecting it.

A sound was first passed into the bladder; an incision was then made into the membranous part of the urethra, and a director introduced into the bladder; when the staff was withdrawn. A bistoire cachée, somewhat in the form of a catheter, was then introduced along the director; when the latter was removed, and the bistoire carried upwards until it pointed at the superior and anterior part of the bladder. An incision was made through the external integuments on the point of the bistoire, but which was not continued into the bladder: the division of the latter was effected by thrusting forward the cutting part of the bistoire cachée, so that its point passed out through the opening already made in the external integuments. The wound was then enlarged downwards by a common probe-pointed bistoury, fitting a groove in the bistoire cachée. The stone was encysted, and it was necessary to separate it with the finger before the application of the forceps. A catheter of elastic gum was then passed through the lower opening into the bladder, and retained there, to prevent accumulation and extravasation of the urine, until the superior wound had healed.

---

*Mode of checking Hæmorrhage consequent on the Extraction of Teeth.*—Mr. Cullen, of Sheerness, recommends the following method for the treatment of the above frequent, and sometimes serious, accident:—

"Take a small, fine, vial cork, of a size adapted to the socket whence the tooth has been extracted and the hæmorrhage proceeds. Then, with a small dossil of lint, wet with aqua styptica, and put on the smallest end of the cork, push the cork into the bleeding orifice, pressing it firmly in, till it be, as it were, wedged in the socket; and keep it there as long as may be necessary, desiring the patient to press against it with the teeth of the opposite jaw till the bleeding be stopped, which it is almost instantly. This acts as a tourniquet, and gives you time to use whatever other means you may deem requisite; but it is seldom that any thing else is required."

---

We expect much valuable addition to our knowledge respecting *materia medica*, from a convention that has been made by the principal physicians of the United States, for the purpose of forming *an American Pharmacopœia*. The several Colleges of Physicians and Surgeons, Medical Schools and Societies, have united in the undertaking; and it has been determined that a convention should be held in each of the four grand divisions of the United States, to be composed of delegates from the colleges, &c.; that each district convention should form a Pharmacopœia, and elect delegates to meet in general convention in the city of Washington, on the 1st of January, 1820; when the general convention will proceed to form the national work from the district convention Pharmacopœias. The different regions of the United States produce many indigenous plants, possessing powerful and important medicinal properties, which merit being more generally known, and more attentive examination, than they have hitherto obtained; and we feel much pleasure in perceiving that such an object has been undertaken in a manner that promises to be productive of so much general benefit; which cannot fail to be the case when superintended by such men as Drs. Mitchell, Hosack, Rodgers, Stearns, Watts, Beck, Spalding, Post, and Stevens.

The formation of a *Code of Medical Ethics*, or institutes and precepts for regulating the professional intercourse and conduct of physicians and surgeons, (to be effected by the same regulations as those proposed for the Pharmacopœia,) has also been proposed; from considering that it would be a mean of exalting the medical character of their country, and have a tendency to prevent frequent misunderstandings among medical men in their professional intercourse.

---

*Some Observations on the alliaceous Odour of White Arsenic;* by Dr. Paris.*—After the various controversies upon the subject of arsenical tests, it is not a little singular that the discordance which exists in the different chemical works of authority, upon one of the most important characters of arsenious acid, should have escaped animadversion. *Does the arsenious acid, when volatilized, yield any alliaceous or perceptible odour?* The fact is, that, unless the arsenical vapour be deoxidized by the presence of some body which has a powerful affinity for oxygen, it is perfectly inodorous, the alliaceous, or garlic-like, smell being wholly confined to *metallic* arsenic in a state of vapour: such a deoxidation takes place when the arsenious acid is thrown upon ignited charcoal, or when heated in contact with those metallic bodies which readily unite with oxygen,—as *antimony, tin*, &c. It is stated by Orfila, and other chemists, that, if it be projected upon heated copper, the alliaceous odour is evolved. This certainly takes place if the copper be in a state of ignition; for, at that temperature, its affinity for oxygen enables it to reduce the arsenious acid: but, if a few grains of this substance be heated on a plate of copper, by means of a spirit-lamp or a blow-pipe, no odour is perceptible; for the whole of the acid is dissipated before the copper acquires a sufficiently exalted temperature. If the arsenious acid be heated on a plate of zinc, the smell is not evolved until the zinc is in a state of fusion. If, instead of these metals, we employ in our experiments gold, silver, or platina, no alliaceous smell whatever is produced."

---

* *Journal of Science and the Arts*, No. xii.

THE *Cancer Institution* is removed to No. 28, Gerard-street, Soho; where Mr. SAMUEL YOUNG, the surgeon to that Institution, now resides, for the purpose of more perfectly fulfilling the objects of that establishment; as he will thus be enabled to illustrate the Lectures, which he will shortly deliver on that disease, by cases on the spot, and also furnish students with ample opportunities of witnessing the appropriate application of the compress and roller, and of acquiring themselves an ability to employ them with the necessary accuracy. We may now hope to see the beneficial influence, which was expected to arise from that Institution, more precisely determined. We shall give our readers further notice of these lectures, and, perhaps, be enabled to supply them with some clinical reports on this subject.

---

*Prize Questions.*—"*Is there any doubt respecting the existence of idiopathic fever?*" is a question proposed by the Medical Society of Paris; for the best reply to which, a prize of the value of 300 francs will be awarded in December 1819. The Memoirs are to be written in French or Latin, and sent (free of expense) to M. NACQUART, the general secretary of the Society, Rue Sainte-Avoie, No. 39, before the 1st of November.

One of the questions proposed the last year by the Medical Society of Paris—"*To determine the nature, causes, and the treatment, of internal hæmorrhages from the uterus, arising during pregnancy, in the course of parturition, and after delivery,*"—not having been treated in a manner perfectly satisfactory to the learned commissioners appointed to examine the Memoirs, is again proposed for the current year. The premium, and other circumstances respecting the mode of proceeding in order to contest it, as above.

The Medical Society of the Department of the Eure has proposed the following:—"*To determine the nature, character, causes, differences, and the treatment, of ascites.*" The prize consists in a gold medal of the value of 200 francs; and a silver medal will be presented to the author of the Memoir which shall be considered next in value to the former. The Memoirs, written in French or Latin, are to be sent (free of expense) to M. L. H. DELARUE, Pharmacien à Evreux, secretary of the Society, before the 1st of August, 1819.

---

THE great mass of recent medical publications from different parts of the world, now before us, yet unnoticed in our Journal, amongst which are several works on subjects of the highest interest and practical importance, obliges us to point out, in a general way, to our readers, some of those which are worthy of their attention, but of which (at present, at least,) we cannot give a regular analysis. We desire it to be understood that, although in the critical department of our Journal our notice of works will bear a relation to their chronological order, yet, on occasions like the present, we must select for immediate consideration those that appear to contain matter of the greatest novelty and direct practical importance.

We shall first point out a translation of the excellent article on *Gout*, in the Dictionnaire des Sciences Medicales, by M. Guilbert, to which a sort of commentary is added by the translator.* This is a valuable addition to English medical literature, as it contains some observations and remarks of importance, in addition to those contained in our latest and best original work on the subject—that of Dr. Scudamore.

The eagerness with which biography of eminent persons is universally perused, has produced many prolix disquisitions from speculative observers: some attributing it to the gratification of curiosity; others, to sentiments forming the principles of Hobbism; and a third class, perhaps more properly, to the excellent theme it affords to the text of Horace:—

"——simul et jucunda et idonea dicere vitæ,

Lectorem delectando simul atque monendo."

This union of agreeable with useful information is particularly evinced in the Memoirs of the living Members of the London College of Physicians; and the general interest they have excited appears to have been consonant with the real importance of the subject, since a second edition of those Memoirs has already been required.† A choice selection of medicinal formulæ, collected apparently both from their public and private practice, is added to the present edition, which will render it of increased value to professional readers; especially to those who, either from want of accurate knowledge in chemistry or practice in the art of prescribing, require a guide to direct them in the mode of exhibiting active remedies with safety and precision.

Second editions of Dr. Armstrong's works on *Scarlatina, Typhus*, and *Puerperal Fever*, have also recently appeared, which contain some useful additional observations, and a further development of the author's opinions.

---

* *Practical Researches on the Nature, Cure, and Prevention of Gout, in all its open and concealed Forms; with a Critical Examination of some celebrated Remedies and Modes of Treatment employed in this Disease.* By James Johnson, Esq. Surgeon to his Royal Highness the Duke of Clarence; author of the "Influence of the Atmosphere on the Health and Functions of the Human Frame," &c.—8vo. pp. 105. Highley and Son, 1818.

† *Authentic Memoirs, biographical, critical, and literary, of the most eminent Physicians and Surgeons of Great Britain; with a choice Collection of their Prescriptions, an Account of the Medical Charities of the Metropolis,* &c.—8vo. pp. 562. Second edition, enlarged. Sherwood and Co. 1818.

A translation of Orfila's Elements of Chemistry is also an useful addition to the English medical library.* This work will be comprised in two volumes, the first of which has only hitherto appeared, which treats of mineral chemistry. The second will consist of the chemistry of animal and vegetable substances, and will contain a number of new and highly interesting experiments.

We would especially direct the attention of our readers to a work called *British Field Sports*,† because of the observations and judicious remarks that it contains respecting the *diseases of dogs*, especially the *rabies* of that animal. We need not point out the importance of knowledge on this subject to medical practitioners, since a little reflection will render it obvious to those who may not already be convinced of it. The parts of the work to which we have alluded possess forcible claims on their attention, since, if Mr. Blane's Treatise be excepted, there did not previously exist a scientific history of the diseases of the dog and their appropriate mode of treatment. Some of our country readers, who have good horses, vigorous health, and leisure, and who are inclined to the study of nature and the habits of animals as well as of books, will derive from this work a species of information and pleasure that they can hardly anticipate. It evinces great accuracy and extent of observation, and a philosophic disposition of mind in the learned author.‡

---

* *Elements of Modern Chemistry; comprehending the latest Discoveries which have been made in this Branch of Science; principally for the use of Students.* By M. P. Orfila, Quarterly Physician to his Majesty Louis XVIII. &c.; improved by the addition of a copious Index and Glossary. Illustrated by plates. 2 vols. —8vo. Cox and Son, 1819.

† *British Field Sports; embracing Practical Instructions in Shooting, Hunting, Coursing, Racing, Cocking, Fishing, &c.; with Observations on the Breaking and Training of Dogs and Horses,* &c. &c. By William Henry Scott.—8vo. pp. 605. Sherwood and Co. 1818.

‡ We suspect W. H. Scott to be a fictitious name: the peculiar originality of manner evident in this work would lead us to attribute it to a gentleman already well known to the public by his zoological treatises. It is somewhat remarkable that two of the English works that have been read with the greatest pleasure were published under fictitious names; and we doubt not but that sportsmen will peruse British Field Sports with as much delight as metaphysicians, critics, and moralists, have enjoyed in the contemplation of the *Anatomie of Melancholie* and *Tristram Shandy*. If Mr. J. L. be the author of this work, we do not see why he should not acknowledge it: an excellent philosopher, who was also one of the greatest generals of ancient Greece, did not consider the same subject unworthy of his pen; and Bacon treated of the arrangement of a garden amongst the matters comprised in his immortal *Essays*.

On speaking of the diseases of brute animals, we must not neglect to point out, as worthy of the attention of our readers, the Treatise of Mr. Wilkinson on Tetanus, and the epidemic catarrhal disease affecting horses.* But this work, from the peculiar interest of its principal subject with respect to medicine in general, which is treated in a manner that forcibly evinces the benefits that will ensue from the establishment of the Veterinary College, will engage our particular attention at a future period. We shall at present, however, observe, that a preternaturally dark appearance of the spinal marrow, and signs of inflammation of its membranes and of those of the brain, were discovered on dissection after death in several instances.

The first part of the work of Professor Meckel on *Pathological Anatomy*† will be contemplated with peculiar interest. It is a work deduced from the labours of half a century, and will comprehend all the natural and acquired derangements of figure, as well as *intimate structure*, that have come under the observation of the author. Much additional light will probably be thrown on this subject, by the collation of deviations from the ordinary state of the organs of the human body with analogous appearances in brute animals; an object that the Professor's favourite pursuits will enable him to accomplish in an able manner. We shall transcribe the following description of a species of deviation from the natural structure of the heart, of frequent occurrence, which will be interesting in itself, and serve also as a specimen of the author's mode of description:—

"Cor est juvenis 14 annorum, morbo coeruleo exstincti. Valvulorum septum nullo modo, ventriculi contra ipsi summopere à norma recedunt. Sinister cum aorta infra solitam capacitatem contractus, dexter contra una cum arteria pulmonali summopere dilatatus. Vitium primarium in parte cordis venosa hæret, ubi foramen ovale modo fere inaudito apertum vides. Memorabile, quod valvula Eustachiana s. foraminis ovalis *anterior* fere omnino evanuerit. Ex cordis sinistri atque aortæ contractione, dextri contra et arteriæ pulmonalis summa dilatatione valvulæ Eustachianæ habitu depauperato, et singulari valvulæ foraminis ovalis s. valvulæ f. o. posterioris quæ itidem minimæ est haud sursum spectat, foraminis ovalis fundo infixa, sed, ejusdem ambitus parti sinistræ adhærens, margine libero, concavo dextrorsum vertitur, recte, ni fallor, in

* *A Treatise on two of the most important Diseases which attack the Horse—Locked Jaw and Tetanus, and the Epidemic Disease, or Catarrhal Affection*, &c. &c. By William Wilkinson, Veterinary Surgeon, of Newcastle.—4to. pp. 212. Longman and Co. 1818.

† *Tabulæ Anatomico-Pathologicæ, modes omnes quibus partium Corporis Humani omnium forma externa atque interna à norma recedit, exhibentes.* Auctore, J. F. Meckel. Fasciculus primus. Cor. cum Tab. viii. fol.—Lipsiæ, apud Gleditsch; et Londini, apud Treuttel et Würtz, Soho-square.

4

hoc casu sanguinem haud ex cavo cordis dextro in sinistrum, sed via contraria, ex sinistro in dextrum transiisse, concluserim."

We must also point out as one of those to be, at least temporarily, set aside, but which, at the same time, we must particularly urge on the attention of our readers,—the work of Dr. Wenzel on the diseases of the uterus;* which, although it does not contain any very important novelties of observation or opinions, yet it will be a valuable addition to the library of the physician; especially to that of those much engaged in the treatment of the diseases of females. The principal morbid affections of the uterus, particularly those from altered structure, are treated in a full and judicious manner. The sections, "Prufung den Heilversuche der Induration und des carcinomates geschwenigen Zustandes an dem Uterus," and "Prufung den Heilversuche der Induration und des carcinomates geschwenigen Zustandes an dem Uterus, durch die anwendung ausserlich und innerlich gebranckter azneikorper," are evidently deduced from the results of long experience and very extensive observation. The work is illustrated by twelve copperplate engravings, and as many outlines only of the same figures, executed in a manner that shows the utmost extent to which graphical representation of morbid anatomy can be carried. But we do not consider that these contribute in any considerable degree to the value of the work; since all attempts to convey adequate ideas of animal structure, and to supply the want of morbid dissections, are totally futile.

We would direct the attention of our readers to a French translation of the Treatise of Mr. Hodgson on Diseases of the Arteries and Veins;† for, at the same time that it shows the liberal spirit of our neighbours, worthy of more general imitation, they will find in it much additional information, of considerable value, in the notes of the translator. We shall shortly furnish our readers with some of the results of the researches of this distinguished anatomist and physiologist, when we take into consideration an original Memoir which he has himself published on Inflammation of Veins.

---

* *Uber die Krankheiten des Uterus;* von Carl Wenzel, der arznei und Wundarzneiwissenschaft Doctor, &c. &c.—Mainz, bei F. Kupferberg; und London, bei Treuttel und Würtz, Soho-square. 1816. Fol. pp. 290. Mit Zwölf Kupfer und eben so vielen Linear tafeln.

† *Traité des Maladies des Artères et des Veines, &c.; traduit d'Anglais, et augmenté d'un grand nombre de Notes,* par Gilbert Breschet, Docteur en Médecine, Prosecteur à la Faculté de Médecine de Paris, Premier Aide de Clinique Chirurgicale à l'Hôtel Dieu, &c. &c. 2 tom. 8vo.—Paris, 1819; et se trouve à Londres, chez Treuttel et Würtz, Soho-square.

### REPORT OF DISEASES.

IT was observed by SYDENHAM, that "cold destroys a much greater number of mankind than the combined ravages of war, famine, and pestilence;" and it is from conviction of the truth of this statement, that the physician feels the weight of his cares somewhat alleviated, now that the season has passed away in which that cause of destruction especially exists. But, whilst the temperature of the atmosphere is below 60° or 65°, there are frequent and numerous states of the system in which cold will act directly as a morbific cause on the animal economy. We have, therefore, yet occasion to witness the prevalence of diseases arising from this agent. This has been observed during the late period especially in children, amongst whom inflammation of the respiratory organs has been of frequent occurrence, and has commonly appeared in the form of *croup*. We need not now dwell on the ordinary nature of this disease, since it occupied our attention in the last Report. But there is an affection evincing several of the characteristics of that malady, which some authors have termed spasmodic croup, which occurs during the prevalence of the former, but differs from it in not being accompanied with symptoms so decisively indicative of the existence of inflammation, and in not often proving fatal in its termination. We have been induced, from repeated observation at different periods, to attribute many cases of this affection to the influence of the imagination. The whole of the rest of the children of the same family are not unfrequently affected in this manner simultaneously, within a few days after one of them had fallen sick of croup, under circumstances which has commonly led the parents to suppose the affection to be contagious; from the latter subjects of it not having been exposed to cold, but when, on the contrary, from the alarm excited by the illness of the first patient, they have been cautiously confined to a warm room: but this, it must be remarked, has been that of the former patient. This affection has commonly been observed in delicate children of great sensibility and irritability of habit, or, as it is frequently termed, of the scrofulous diathesis, because they are also particularly disposed to that form of disease. The above-mentioned notion will, probably, appear devoid of solid foundation to many persons, particularly to those who have not had extensive opportunities for witnessing the diseases of children, and who have not diligently perused the works of the best of the older authors, which are full of instances of the powerful influence of the imagination of children in the production of various species of disease. The ancients, from the time of ASCLEPIADES, who first urged its importance, paid great attention to the influence of the mind, as a cause as well as a remedy of disease: but physicians of the present age have almost entirely resigned such views of pathology and therapeutics to empirics. Whether what has been stated, or particular habit of body, be the cause of it, it is certain that there is a form of croup, unaccompanied either with fever, much general excitement, expectoration of viscid phlegm, or inflammatory redness about the upper part of the pharynx; and in which *the respiration is more free and less shrill and sonorous during sleep* than when the patient is awake; which may be relieved without having recourse to blood-letting, and the other general sedative measures mentioned in the last Report. These cases have usually been promptly removed by counter-irritants; as the application of *hot* water to the feet, purgatives of calomel and jalap, a vesicatory to the throat, and as much ipecacuanha as could be borne without causing vomiting.

We are induced to believe that the above etiological remarks are also applicable, under certain circumstances, to *phthisis*; but on this point we shall quote the opinions of a philosopher, whose works merit a place in every medical library, from the great number of interesting facts and reflections they contain respecting the influence of the moral on the physical phenomena of the animal economy.

"Simon Thomas," dit MONTAIGNE, "estoit un grand medecin de son temps. Il me souvient que me rencontrant un jour à Thoulouse chez un riche vieillard pulmonique, et traitant avec luy des moyens de sa guerison, il luy dist, que c'en estoit l'un, de me donner occasion de me plaire en sa compagnie; et que fichant ses yeux sur la frescheur de mon visage et sa pensée sur cêtte allegresse et vigueur, qui regorgeoit de mon adolescence; et remplissant tous ses sens de cet estat florissant en quoy j'estoit lors; son habitude s'en pourroit amender: *Mais il oublioit à dire, que la mienne s'en pourroit empirer aussi*"—(*Essais*, liv. i. chap. 20.)

Thoracic disorders in adults evince the same characters as those noticed in the last Report; which remark will also apply to the generality of the diseases to which they are at present especially disposed.

We need make no particular observations respecting *typhous fever*, as there is nothing remarkable in its present character, and we fear is not more prevalent than it will ever be in our metropolis.

Whooping-cough is of rather frequent occurrence amongst children. Except in a few cases, this complaint may, in the delicate habits of children educated in the city, be safely, and indeed most effectually, treated by counter-irritants. It has generally been found that the internal exhibition of ipecacuanha with rhubarb or jalap, and the production of a rash on the surface of the chest by means of the tartar-emetic ointment, has soon removed this disease. The tartar-emetic ointment is preferable to any of the stimulating embrocations usually employed; such as oil of amber, tincture of cantharides diluted, volatile alkali, &c.

---

### MONTHLY CATALOGUE OF MEDICAL BOOKS.

### METEOROLOGICAL JOURNAL,

**By Messrs. William Harris and Co. 50, Holborn, London.**

***From the 20th February to the 19th March, 1819, inclusive.***

| Day of Month. | Moon. | Rain gauge | Therm. | | | Barom. | | De Luc's Hygrom. Dry | Damp | | Wind. | | Atmospheric Variation. | | |
|---|---|---|---|---|---|---|---|---|---|---|---|---|---|---|---|
| Feb. | | | | | | | | | | | | | | | |
| 20 | | | 41 | 45 | 40 | 29·79 | 29·81 | | 10 | 11 | W | W | Fine | | |
| 21 | | | 43 | 47 | 40 | 29·80 | 29·78 | | 12 | 13 | WSW | W va. | Fine | Rain | Clo. |
| 22 | | | 42 | 46 | 35 | 29·86 | 29·97 | | 11 | 13 | N | SW | Fine | Rain | Clo. |
| 23 | | | 40 | 44 | 33 | 29·90 | 30·00 | | 12 | 11 | N | N | Clo. | Fine | |
| 24 | ● | | 37 | 35 | 28 | 29·56 | 29·63 | | 9 | 10 | WNW | N | Fine | Clo. | Fine |
| 25 | | | 34 | 38 | 34 | 29·80 | 29·82 | | 10 | 11 | N | NW | Fine | | |
| 26 | | | 34 | 38 | 35 | 29·60 | 29·45 | | 9 | 11 | NW | NW | Fine | Clo. | Rain |
| 27 | | | 41 | 44 | 49 | 29·43 | 29·43 | | 12 | 13 | SSW | SSE | Clo. | | Rain |
| 28 | | | 49 | 51 | 34 | 29·35 | 29·35 | | 13 | 14 | SSE | E | Rain | | |
| Mar. | | | | | | | | | | | | | | | |
| 1 | | | 34 | 38 | 38 | 29·28 | 29·34 | | 14 | 13 | E | NE | Rai.& Snow | Rain | |
| 2 | | ,28 | 40 | 39 | 37 | 29·35 | 29·52 | | 14 | 13 | NE | NE | Rain | Clo. | |
| 3 | ☽ | ,11 | 39 | 40 | 39 | 29·63 | 29·83 | | 11 | 11 | E | E | Clo. | | |
| 4 | | ,08 | 41 | 44 | 38 | 29·91 | 29·96 | | 11 | 12 | NE | ENE | Fine | Clo. | Rain |
| 5 | | ,02 | 40 | 44 | 40 | 30·01 | 29·96 | | 13 | 12 | NE | NNE | Clo. | | Rain |
| 6 | | | 42 | 45 | 38 | 29·92 | 30·02 | | 13 | 12 | ENE | ENE | Rain | Fine | |
| 7 | | | 40 | 44 | 39 | 30·09 | 30·13 | | 11 | 12 | NE | NE | Clo. | | Rain |
| 8 | | | 40 | 42 | 38 | 30·08 | 30·01 | | 13 | 13 | NE | NE | Clo. | | |
| 9 | | | 40 | 43 | 39 | 30·07 | 30·13 | | 12 | 10 | S | SW | Clo. | | Fine |
| 10 | | | 43 | 45 | 40 | 30·11 | 30·05 | | 13 | 12 | NW | N | Clo. | | Fine |
| 11 | ○ | | 45 | 50 | 41 | 30·07 | 30·11 | | 13 | 13 | NW | NW | Fine | | |
| 12 | | | 47 | 51 | 42 | 30·17 | 30·24 | | 12 | 13 | NW | NW | Fine | | |
| 13 | | | 47 | 50 | 40 | 30·30 | 30·31 | | 13 | 11 | NW | NE | Clo. | | |
| 14 | | | 44 | 48 | 38 | 30·29 | 30·24 | | 12 | 13 | NW | SSE | Fine | | |
| 15 | | | 43 | 50 | 43 | 30·16 | 30·10 | | 13 | 13 | SE | SE | Fine | | Rain |
| 16 | | | 48 | 52 | 44 | 30·07 | 30·03 | | 12 | 11 | SE | NW | Fine | | |
| 17 | | | 47 | 53 | 37 | 30·11 | 30·21 | | 9 | 7 | NE | NNE | Fine | | Clo. |
| 18 | | ,02 | 41 | 47 | 41 | 30·24 | 30·04 | | 6 | 8 | NW | W va. | Fine | | |
| 19 | ☾ | ,08 | 45 | 49 | 41 | 29·61 | 29·39 | | 12 | 13 | SW | NW | Rain | Fine | Rain |

The quantity of rain fallen in the month of February, is 1 inch and 52-100ths.

